# Arachidonic acid triggers [Ca^2+^]_i_ increases in rat round spermatids by a likely GPR activation, ERK signalling and ER/acidic compartments Ca^2+^ release

**DOI:** 10.1371/journal.pone.0172128

**Published:** 2017-02-13

**Authors:** Joaquin Paillamanque, Ana Sanchez-Tusie, Emerson M. Carmona, Claudia L. Treviño, Carolina Sandoval, Francisco Nualart, Nelson Osses, Juan G. Reyes

**Affiliations:** 1 Instituto de Química, Pontificia Universidad Católica de Valparaíso, Valparaíso, Chile; 2 Departamento de Genética del Desarrollo y Fisiología Molecular, Instituto de Biotecnología, Universidad Autónoma de México, Cuernavaca, México; 3 Departamento de Biología Celular, Facultad de Ciencias Biológicas, Universidad de Concepción, Concepción, Chile; Cinvestav-IPN, MEXICO

## Abstract

Arachidonic acid (AA), a compound secreted by Sertoli cells (SC) in a FSH-dependent manner, is able to induce the release of Ca^2+^ from internal stores in round spermatids and pachytene spermatocytes. In this study, the possible site(s) of action of AA in round spermatids, the signalling pathways associated and the intracellular Ca^2+^ stores targeted by AA-induced signalling were pharmacologically characterized by measuring intracellular Ca^2+^ using fluorescent Ca^2+^ probes. Our results suggest that AA acts by interacting with a fatty acid G protein coupled receptor, initiating a G protein signalling cascade that may involve PLA2 and ERK activation, which in turn opens intracellular ryanodine-sensitive channels as well as NAADP-sensitive channels in acidic intracellular Ca^2+^ stores. The results presented here also suggest that AMPK and PKA modulate this AA-induced Ca^2+^ release from intracellular Ca^2+^ stores in round spermatids. We propose that unsaturated free fatty acid lipid signalling in the seminiferous tubule is a novel regulatory component of rat spermatogenesis.

## Introduction

The functional relationship between germ and Sertoli cells (SCs) in the mammalian seminiferous tubules takes place through adhesion molecules and molecules secreted to the extracellular space in the adluminal compartment [1–3]. The precise composition of the extracellular environment of germinal cells in the seminiferous tubules remains unknown, but it is well established that SCs secrete molecules that are crucial for the spermatogenic process to proceed. Among several molecules, SCs release arachidonic acid (AA) and some of its metabolites in a FSH-regulated manner [4]. In a SC-derived cell line (TM4) the activation of CD95 (Fas), a pathway that participates in spermatogenic cell apoptosis [5,6] can activate cytosolic PLA2 and AA release [7]. Furthermore, AA has been shown to be able to activate glycolytic lactate production in SCs in an autocrine loop [8]. These evidences strongly suggest that AA is part of the cell-cell signalling regulatory network of spermatogenesis; a role that is also emphasized by the large effect that AA has as a nutrient on animal fertility [9], and by the infertility effects seen after knocking-down a group VI PLA2 isoform expressed in mice testes [10].

In a previous work, we showed that AA and other unsaturated fatty acids (UFA) were able to release Ca^2+^ from intracellular Ca^2+^ stores (ICaS) in a dose-dependent manner in pachytene spermatocytes and round spermatids [11]. In this work, we provide evidence that agonists for GPR120 but not for GPR40 (both GPRs activated by medium and long chain FAs) were able to induce an increase in intracellular Ca^2+^ concentration ([Ca^2+^]_i_) in spermatogenic cells in the nominal absence of external Ca^2+^. This Ca^2+^ was released from ICaS, similarly to the effects of AA. Immunohistochemistry showed that GPR120 was present in spermatogenic cells. Using pharmacological effectors of downstream signalling pathways in these cells, we determined that the AA effect of releasing Ca^2+^ from ICaS was sensitive to ERK and PLA2 inhibition. Furthermore, modulators of AMPK and PKA affect this AA-induced intracellular Ca^2+^ release in round spermatids. Both the cADPribose-sensitive channel inhibitor ryanodine and the NAADP-sensitive channel inhibitor NED19 were able to slow the release of Ca^2+^ elicited by AA. We also showed that round spermatids presented NAADP-sensitive ICaS, which is consistent with the inhibitory action of NED19 on AA-induced Ca^2+^ release from ICaS. Thus, we provide evidences to support the hypothesis that UFAs can be part of the cell-cell signalling mechanisms in seminiferous tubules, leading to the control of spermatogenic cell differentiation or death by SCs in the testes.

## Materials and methods

### Animals

Adult (40–60 days old) male Sprague–Dawley rats were acquired from the Animal Facility in the Faculty of Sciences University of Valparaiso. The rats were housed under a 12L:12D cycle with water and rat chow *ad libitum*. Adult rats were maintained in groups of four animals per cage. Two rats were chosen at random and their testicles were used to isolate round spermatids as described below.

### Ethical statement

All the experiments were conducted in accordance with the guidelines outlined by the Consortium for Development in the Guide for the Care and Use of Agricultural Animals in Agricultural Research and Teaching and by the National Research Council. All experimental protocols were reviewed and approved by the Chilean National Fund for Science and Technology (FONDECYT) and the Ethics Committee of the Pontificia Universidad Católica de Valparaíso (EC-PUCV-10/2013). None of the authors served on this committee.

### Rat pachytene spermatocytes and round spermatid isolation

The testicles were obtained from adult (60 day old) male Sprague-Dawley rats. The rats were exposed to CO_2_ and then killed by cervical dislocation. Rat spermatogenic cell populations were isolated using velocity sedimentation separation in a 2–4% BSA gradient, as described by Romrell *et al*. (1976) [12]. The round spermatid fraction (92±4% purity) was identified both by its size as well as by its typical nuclear aspect after staining with Hoescht 33342 [13].

### Reagents

Fura-2 acetoxy methyl ester, 2',7'-Bis-(2-carboxyethyl)-5-(and-6)-carboxyfluorescein acetoxy methyl ester (BCECF) and Hoescht 33342 were purchased from Molecular Probes (Life Technologies, USA). Ryanodine, GPR40 agonist (PPMAPP, 3-(4-(((3-(Phenoxy)phenyl)methyl)amino)phenyl) propanoic acid), ERK Activation Inhibitor Peptide I, ERK Activation Inhibitor Peptide II (5-(2-Phenyl-pyrazolo[1,5-a]pyridin-3-yl)-1H-pyrazolo[3,4-c]pyridazin-3-ylamine), GPR120 agonist III (3-(4-((4-Fluoro-4′-methyl-(1,1′-biphenyl)-2-yl)methoxy)-phenyl)propanoic acid), GPR40 agonist II (3-(4-((2,6-Dichloropyridin-4-yl) ethynyl)phenyl)propanoic acid), okadaic acid (OA, 9,10-Deepithio-9,10-didehydroacanthifolicin, cantharidin (Canth, (3aα,4β,7β,7aα)-Hexahydro-3a,7a-dimethyl-4,7-epoxyisobenzofuran-1,3-dione), cyclosporin A and RU360 were purchased from Calbiochem (Merck Chem. Co., Chile). Arachidonic acid, dantrolene, caffeine, 2-APB (2-aminoethyl diphenyl borate), enzymes, salts and buffers were obtained from Sigma-Aldrich (St. Louis, MO, USA). Grifolic acid (2,4-Dihydroxy-6-methyl-3-[(2*E*,6*E*)-3, 7,11-trimethyl-2,6,10-dodecatrien-1-yl]-benzoic acid), PT1 (2-Chloro-5-[[5-[[5-(4,5-Dimethyl-2-nitrophenyl)-2-furanyl]methylene]-4,5-dihydro-4-oxo-2-thiazolyl]amino]benzoic acid), H89 (*N*-[2-[[3-(4-Bromophenyl)-2-propenyl]amino]ethyl]-5-isoquinolinesulfonamide dihydrochloride) and transNED19 ((1*R*,3*S*)-1-[3-[[4-(2-Fluorophenyl)piperazin-1-yl]methyl]-4-methoxyphenyl]-2,3,4,9-tetrahydro-1*H*-pyrido[3,4-*b*]indole-3-carboxylic acid) were obtained from Tocris Bioscience (R&D Systems, Gene X-Press, Chile). All the agonists, activators or inhibitors used in this work are listed in Table 1 together with their main target and effect, as well as their source and representative references.

**Table 1:** Pharmacological effectors.

PHARMACOLOGICAL EFFECTORS: TARGETS, SOURCES AND REFERENCES			
Name	Target and Effect	Source	References
GPR120 agonist III ^(1)^	GPR120 agonist	Calbiochem	[14]
GPR40 agonist II ^(1)^	GPR40 agonist	Calbiochem	[15]
Grifolic acid ^(1)^	GPR120 agonist	Tocris Bioscience	[16]
PPMAPP ^(1)^	GPR40 agonist	Calbiochem	[17]
Mastoparan X ^(3)^	G protein analogue; Membrane permeant	Tocris Bioscience	[18–20]
SCH202676 ^(3)^	GPR/G protein binding inhibitor (sulfhydryl reagent); Membrane permeant	Tocris Bioscience	[21,22]
SP 4–11 ^(3)^	Substance P-peptide inhibitor of G protein binding to GPR; Membrane permeant	Tocris Bioscience	[23,24]
Akt Inhibitor 4 ^(S3A)^	Akt inhibitor; Membrane permeant	Calbiochem	[25]
Akt Inhibitor 5 ^(S3A)^	Akt inhibitor; Membrane permeant	Calbiochem	[25]
ERK inhibitory peptide I ^(S3A)^	ERK inhibitor; Membrane permeant	Calbiochem	[26]
ERK inhibitory peptide II ^(S3A)^	ERK inhibitor; Membrane permeant	Calbiochem	[26]
Wortmannin ^(S3A)^	PI3K inhibitor; Membrane permeant	Calbiochem	[27]
Bisindolylmaleimide ^(S3B)^	PKC inhibitor; Membrane permeant	Sigma-Aldrich	[28]
Calmidazolium ^(S3B)^	CaM inhibitor; Membrane permeant	Calbiochem	[29]
Forskolin ^(S3B)^	Adenylyl cyclase activator; Membrane permeant	Calbiochem	[30]
H89 ^(S3B)^	PKA inhibitor (see Lochner and Moolman, 2006 for other targets); Membrane permeant	Tocris Bioscience	[31,32]
KN-62 ^(S3B)^	CaMK inhibitor; Membrane permeant	Calbiochem	[33]
PT1 ^(4B)^	AMPK activator; membrane permeant	Tocris Bioscience	[34,35]
Trifluoperazine ^(S3B)^	CaM inhibitor; Membrane permeant	Sigma-Aldrich	[36]
Cantharidin ^(S3C)^	Protein Pase 1 and 2A inhibitor; Membrane permeant	Calbiochem	[37]
Cyclosporine A ^(S3C)^	Calcineurin inhibitor; Membrane permeant	Calbiochem	[38]
Okadaic acid ^(S3C)^	Protein Pase 1 and 2A inhibitor; Membrane permeant	Calbiochem	[39]
Aristolochic acid ^(S3D)^	PLA2 inhibitor, Membrane permeant	Sigma-Aldrich	[40,41]
U73122 ^(S3D)^	PLC inhibitor; Membrane permeant	Sigma	[42,43]
U73343 ^(S3D)^	Inactive Analogue of U73122; Membrane permeant	Sigma	[43]
Caffeine ^(5)^	RyR sensitizer, cAMP phosphodiesterase inhibitor; membrane permeant	Sigma-Aldrich	[44]
Dantrolene ^(5)^	cADPribose-sensitive channel inhibitor (RyR); Membrane permeant	Sigma-Aldrich	[45]
transNED19 ^(5)^	NAADP-sensitive channel inhibitor; Membrane permeant	Tocris Bioscience	[46]
RU360 ^(5)^	Mitochondrial Ca2+ uptake inhibitor; Membrane Permeant	Calbiochem	[47]
2-APB ^(5)^	IP3-sensitive channel inhibitor; Membrane permeant	Sigma-Aldrich	[48]
Ryanodine ^(5)^	cADPribose-sensitive channel inhibitor (RyR); Membrane permeant	Calbiochem, Tocris Bioscience	[49–51]
NAADP-AM ^(6)^	Two pore channel agonist; Membrane permeant	Synthesized	[52]

Each bracketed number represents a Figure number: (1) GPCR’s agonists; (3) G Protein/GPR effectors; (S3A) MAPK-PI3K-Akt; (S3B) CaM-PKC-PKA-CaMK-AMPK; (S3C) PL inhibitors; (S3D) Protein phosphatases effectors; (5, 6) ICaS Ca^2+^ transport effectors.

### Intracellular Ca^2+^ measurements of rat spermatogenic cells

Cells in suspension (20 x 10^6^ cells/mL) were loaded with 5 μM Fura-2 AM by incubation for 1 h at room temperature under an O_2_ atmosphere. Thereafter, the cells were washed three times in KH-lactate medium (in mM, NaCl: 140; KCl: 4; MgCl_2_:1.6; CaCl_2:_ 0.5; KH_2_PO_4_: 1.6; HEPES 10; DL lactate: 10; pH 7.4; pH balanced with NaOH) and maintained in this media unless stated otherwise. The measurements were performed by adding concentrated cells in suspension (50 μl, to reach 2x10^6^ cells/ml) to a temperature-regulated and stirred spectro-fluorimeter cuvette that contained 2.5 mL of a KH-no Ca^2+^-EGTA-lactate (0.5 mM EGTA, 10 mM DL lactate) solution in a Fluoromax 2 fluorimeter (Jobin-Yvon-Spex, NJ, USA). [Ca^2+^]i was calculated using a ratiometric method as described by [53]. Fura-2 calibration was performed by cell lysis with digitonin (20 μg/mL) and addition of EGTA to 1.5 mM final concentration (Fmin). Fmax was obtained by addition of a CaCl_2_ solution to the digitonin-treated cell suspension giving 3 mM final Ca^2+^ concentration. The N reported is the number of measurements performed in at least three cell preparations with four testicles each. The results are expressed as the mean ± standard error of the mean (SEM).

### Immune histochemistry

The testes of 60 day old Sprague-Dawley rats were removed and submerged immediately for 4 h in Bouin´s solution or modified Davidson solution [54] for immunohistochemistry or immunofluorescence, respectively. Subsequently, holes were punctured in the tunica albuguinea using a needle and the testes submerged again in the fixative fluid for 20 h. They were then washed in PBS for 30 min and cryoprotected with 10%, 20% and 30% (w/v) sucrose in PBS for 4 h, 4 h and overnight, respectively. Testes sections of 12 μm were cut using a microtome cryostat (HM 525, MICROM International GmbH) at -20°C and stored at the same temperature until use. Immunohistochemistry was performed using a Lab Vision^™^ UltraVision^™^ Detection System: anti-Rabbit HRP/DAB (Thermo Scientific, TR-015-HD) according to the manufacturer instructions. Briefly, sections were thawed at room temperature and rehydrated in PBS. Endogenous peroxidase activity was quenched with 10% H_2_O_2_ solution. The sections were washed with PBS-T and blocked with Ultra V Block. These sections were then incubated with anti-GPR120 antibody (H-155: sc-99105, rabbit polyclonal; raised against amino acids 78–232 at the C-terminus of human GPR120, Santa Cruz Biotechnology) at 4 μg/mL in a humid chamber at 4°C overnight, washed, and incubated with biotinylated goat anti-rabbit antibody (1:300). After washing, the sections were incubated with streptavidin-peroxidase, washed, and a mixture of DAB chromogen and substrate was added until a brown colour was visible and the reaction was stopped with water. Sections were counterstained using hematoxylin, dehydrated with alcohol solutions and xylol, and mounted using Entellan. Microscopic observations and picture acquisitions were performed using a Nikon Diaphot Microscopy and Nikon D3000 Camera. Adjustments of brightness and contrast in the images were made using ImageJ software [55].

To perform immunofluorescence staining, thawed slides were washed with a buffer containing 3.5 mM KH_2_PO_4_, 120 mM NaCl, 10 mM Tris, and 8.4 mM Na_2_HPO_4_, pH 7.8 (Tris-P buffer) and subjected to an antigen retrieval protocol in 10 mM citrate buffer pH 6.0 heated for 5 minutes in a microwave. Slides were left 30 minutes at room temperature in the heated antigen retrieval buffer and then washed with Tris-P buffer. Anti-GPR120 antibody (H-155: sc-99105, rabbit polyclonal; raised against amino acids 78–232 at the C-terminus of human GPR120, Santa Cruz Biotechnology) at 8 μg/mL and anti-Proliferating Cell Nuclear Antigen antibody (anti-PCNA, M0879, mouse monoclonal, Dako) at 1:300 dilution were mixed in Tris-P buffer 1% BSA. Slides were incubated in this primary antibody mixture in a humid chamber at room temperature overnight, then washed, and incubated with a secondary antibody mixture containing anti-rabbit IgG antibody conjugated to Alexa Fluor 488 (711-545-152, donkey polyclonal, Jackson ImmunoResearch) and anti-mouse IgG antibody conjugated to DyLight 549 (715-505-150, donkey polyclonal, Jackson ImmunoResearch), both at 1:200 dilution, and Hoechst 33258 at 2 μg/mL in Tris-P buffer 1% BSA. Incubation was performed in a humid chamber at room temperature, and protected from light for 2 hours, and then slides were washed with Tris-P buffer, and mounted. Spectral confocal microscope (Zeiss 780) was used to obtain images.

### Synthesis of acetoxymethyl NAADP

NAADP and NAADP-AM were synthesized from NADP as previously described [56].

### Data and statistical analysis

The initial rate of effector-induced Ca^2+^ entry was estimated by linear regression of the [Ca^2+^]_i_ vs time, recorded between 30 and 200 s after addition of the compounds. Statistical differences between treatment and control rates of [Ca^2+^]_i_ changes were analyzed by applying ANOVA followed by Welch’s t test. When the data was expressed as the percentage increase in [Ca^2+^]_i_ by AA in the absence or presence of different effectors of [Ca^2+^]_i_ homeostatic mechanisms or signalling pathways, or when the F test indicated significant differences in group variances, the comparison between treatment and control was performed using non-parametrical Mann-Whitney analysis.

## Results

### GPR120 agonists and AA increased [Ca^2+^]_i_ in spermatogenic cells

**Fig 1:**
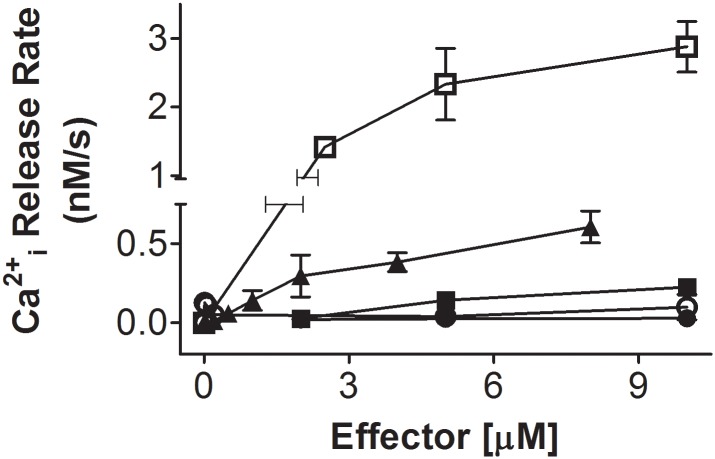
Dose-response curves. Dose-response curves for grifolic acid (□; GPR120 agonist), Arachidonic acid (▲), GPR120 agonist III (■), GPR40 agonist (PPMAPP; ○) and GPR40 agonist II (●)-induced release of intracellular Ca^2+^ in round spermatids vs. their concentrations. Cells were loaded with fura-2 and incubated in KH-lactate-EGTA media without added Ca^2+^ at 33°C. N = 3 for each agonist concentration shown.

Fig 1 shows the dose response curves for the [Ca^2+^]_i_ increase caused by the addition of arachidonic acid (AA), grifolic acid (GA, GPR120 agonist), GPR120 agonist 3, PPMAPP (GPR40 agonist) and GPR40 agonist 2 in round spermatids incubated in KH lactate-EGTA media (see also [11]). Fitting of hyperbolic dose-response curves gave K_0.5_ and Vmax values of 4.6±1.0 μM and 4.3±0.4 nM/s; and 4.2±1.0 μM and 0.8±0.1 nM/s for grifolic acid and AA, respectively. Thus, although it had a similar K_0.5_ to AA, grifolic acid was able to induce approximately a five times faster release of Ca^2+^ from ICaS than AA at saturating concentrations. GPR120 agonist 3 at 5 and 10 μM, although not reaching saturation, was able to induce Ca^2+^ release from ICaS at higher rates compared to GPR40 agonists, but at lower rates compared to GA or AA at similar concentrations. Except at 10 and 50 nM, PPMAPP did not induce a significant increase in [Ca^2+^]i even at 10 μM. GPR40 agonist 2 did not increase significantly the [Ca^2+^]i in round spermatids. These data strongly suggest that AA may be acting through a similar mechanism to the GPR120 agonists, e.g., by activating a G protein-coupled receptor.

The kinetics of grifolic acid-induced intracellular Ca^2+^ release in round spermatids is shown in S1 Fig. The effect of 4 μM AA acid on [Ca^2+^]i was diminished as the GA concentration increased, and at 10 μM GA, it was completely abolished. These results strongly suggest that GA and AA release Ca^2+^ from similar ICaS in round spermatids.

### Expression and distribution of GPR120 in rat testes

**Fig 2:**
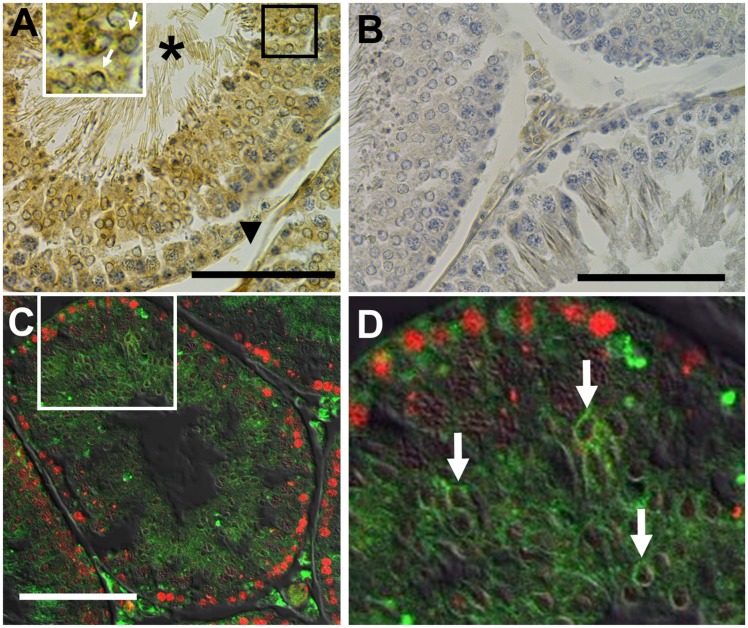
GPR120 immunohistochemistry and immunofluorescence. Representative immunohistochemistry and immunofluorescence images of 60 day old rat testes slices cut in a cryostat using an anti-rat GPR120 rabbit antibody (4 μg/mL) (A, C and D). The secondary antibody and stain developing system was a donkey anti-rabbit antibody coupled to an HRP/DAB system (A and B). A control using rabbit pre-immune serum instead of primary antibody is shown in (B). White arrows point toward spermatids. The asterisk (*) and the black arrow head indicate seminiferous tubule lumen and insterstitium, respectively. The bar shown in Figures A, B and C represents 100 μm. C. Confocal images obtained using as primary antibodies an anti-rat GPR120 rabbit antibody (8 μg/mL) and an anti-PCNA mouse antibody. An anti-rabbit IgG antibody conjugated to Alexa Fluor 488 and anti-mouse IgG antibody conjugated to DyLight 549 were used as secondary antibodies. D. Amplified image of the square shown in C.

To explore the presence of GPR120 in round spermatids as suggested by the pharmacological data and the AA-induced [Ca^2+^]i increase, we utilized an antiGPR120 antibody to perform immunohistochemistry in rat testes. Fig 2A and 2B shows that the antiGPR120 antibody gives a stronger staining toward the luminal side of the seminiferous tubules. Confocal images in Fig 2C and 2D corroborate that GPR120 appears strongly on interstitial cells and also on spermatogenic cells close to the lumen of the tubules. The anti-PCNA antibody shows a well-defined staining on basal cells, presumably, dividing spermatogonia. However, these images cannot discard that part of the GPR120 labelling could be present also on Sertoli cell projections.

### Basal effects of the pharmacological agents on [Ca^2+^]i in round spermatids

In the conditions used in our measurements, round spermatids [Ca^2+^]i progressively increased at an average rate of 0.04 nM/s. As controls, but also to gain information on their actions on ICaS, the pharmacological agents used in this work were first tested for their basal effects on [Ca^2+^]i in round spermatids (S2 Fig). The inhibitor 2-APB (an inhibitor of IP3-sensitive ICaS channels), ERK inhibitory peptide II (Inhibitor peptide I fused to HIV-TAT), KN62 (an inhibitor of CAMK2) and SCH 202676 (a sulfhydryl-reactive compound inhibiting G protein binding to GPRs) induced significant rises in [Ca^2+^]i in these cells. Conversely, TFP (calmodulin inhibitor, but also described as a ryanodine-sensitive channel agonist, [57]) and ArisA (inhibitor of PLA2) were able to significantly decrease the basal rate of [Ca^2+^]_i_ increase in round spermatids. Since the cells are in a media with approximately 5 nM external Ca^2+^, any increase in [Ca^2+^]i can be interpreted as Ca^2+^ release from ICaS. Decreases in the basal rate of [Ca^2+^]i changes can be interpreted as a net decrease in Ca^2+^ release from ICaS, or activation of plasma membrane associated Ca^2+^ efflux.

### Pharmacological evidence that GPR/G proteins are involved in the AA-induced changes in [Ca^2+^]i in round spermatids

**Fig 3:**
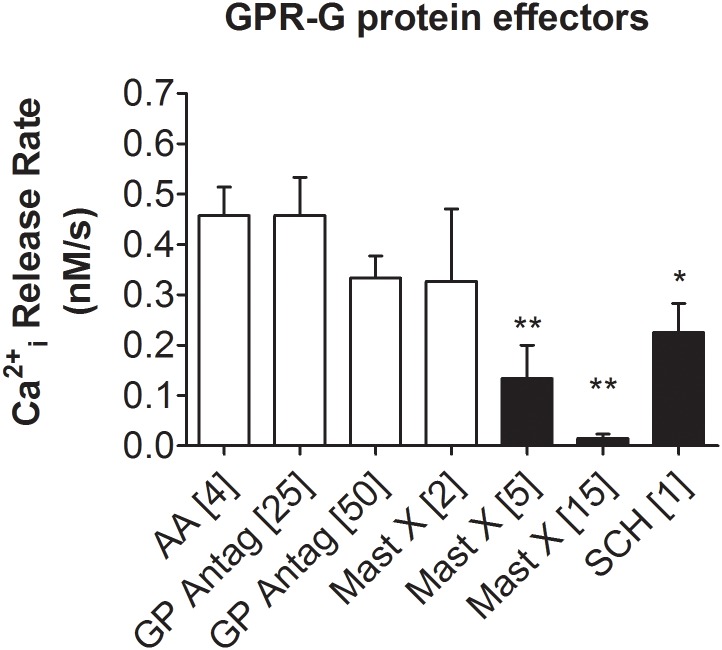
GPR/G protein modulators. Effects of GPR/G-protein modulators on the AA-induced (4 μM) release of Ca^2+^ from intracellular stores. Bracketed numbers in the Figure represent the chemical final concentration in solution. Substance P-G protein antagonist peptide (25 and 50 μM), Mastoparan X (inhibitor of G protein receptor binding, 2, 5 and 15 μM) and SCH202676 (inhibitor of agonist and antagonist binding to G-protein-coupled receptors, 1 μM) were added to the cells in suspension and incubated for 5 min at 33°C before AA addition. Each bar represents the data obtained from three different cell preparations (N = 3). Significance (Black bars): **, p<0.01; *, p<0.05.

In order to explore the possible involvement of G protein-associated pathways activated by AA, we tested the effects of the following compounds: 1) SP 4–11, a Substance P-like hydrophobic peptide claimed to compete with its receptor binding to G proteins used here as a control peptide [23]; 2) mastoparan, a bee-venom derived cationic peptide described to stimulate GTP exchange and activation of G protein but to compete in general for G protein receptor binding [20]; and 3) SCH202676, a sulfhydryl-reactive compound shown to inhibit agonist and antagonist binding to G-protein-coupled receptors [22]. As shown in Fig 3, mastoparan at concentrations of 5 and 15 μM, and SCH202676 at 1 μM were able to induce significant decreases in the AA-induced (4 μM) [Ca^2+^]i increase in round spermatids. Thus, our data are consistent with involvement of G proteins in the signalling associated to the AA-induced changes in [Ca^2+^]i in round spermatids. In rat round spermatids, the commercially available inhibitor for GPR120 (AH7614, Tocris BioScience) induced by itself an increase in [Ca^2+^]i in the absence of AA, discarding its use as an inhibitor of the AA-induced [Ca^2+^] increases shown in these cells. No further testing on the mechanisms of AH7614-induced changes in round spermatids [Ca^2+^]i was performed in this work.

### Pharmacological evidence for signalling pathways involved in the AA-induced changes in [Ca^2+^]i in round spermatids

**Fig 4:**
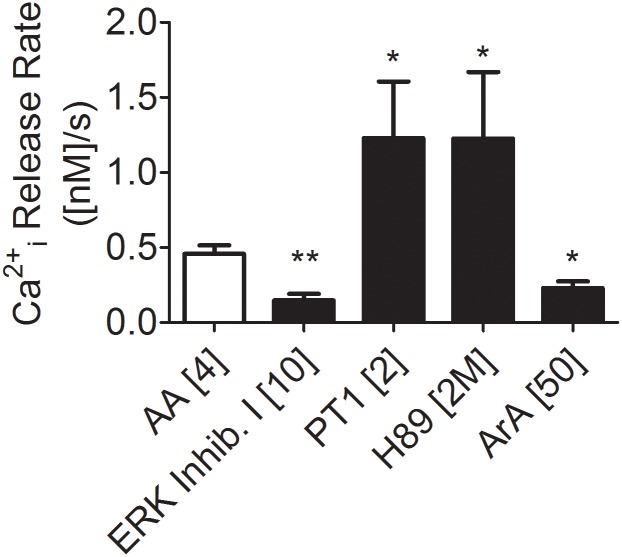
Pharmacological agents with a significant effect on AA-induced increase in [Ca^2+^]i. Consolidated data showing those pharmacological agents that presented a statistically significant effect on AA-induced initial rate of [Ca^2+^]i increase. ERK inhibitory peptide I (10 μM); AMPK activator: PT1 (2 μM); PKA inhibitor: H89 (2 μM); Inhibitor of PLA2: Aristolochic acid (ArA, 50 μM) Significance: *, p<0.05; **, p<0.01. Error bars represent the standard deviation from at least 3 different cell preparations. The effects of all the signalling pathways effectors utilized in this work are shown in (S3A, S3B, S3C and S3D Fig).

In order to explore the signalling pathways associated to AA-induced Ca^2+^ release from ICaS, we utilized ERK inhibitory peptide I, [26] and ERK inhibitory peptide II (Inhibitor peptide I fused to HIV-TAT), Akt Inhibitors 4 [25,58] and 5 [25] and wortmannin, an inhibitor of PI3K [27] (see Fig 4 and S3A Fig). Neither wortmannin nor Akt inhibitors 4 or 5 produced a significant change in the AA-induced Ca^2+^ release from ICaS. Instead, ERK inhibitory peptide I induced a significant decrease in the AA-induced Ca^2+^ release from ICaS. ERK inhibitory peptide II resulted in a large variance and did not produce a statistically significant effect. Since ERK inhibitory peptide II is expected to bind to membranes, it is likely that its free concentration could be comparatively less than inhibitor peptide 1 and also it can be expected to induce membrane structure perturbations in these cells. Regardless, our data suggest that ERK could be involved in the signalling and activating pathways associated with the AA effect on [Ca^2+^]i in these cells.

Since AA-induced [Ca^2+^]i changes could trigger activation of Ca^2+^ signalling in round spermatids, we explored pharmacologically whether calmodulin, CAMK, PKC or PKA could be involved in the AA effect of ICaS release in these cells. For that purpose, we used trifluoperazine (TFP, a non-specific CaM inhibitor; [57]), calmidazolium (CMDZ, a CAM inhibitor; e.g., [29]), KN-62 an inhibitor of CAMKII [33], bisindolylmaleimide (BIM) as a general inhibitor of PKC [28] and forskolin as an activator of adenylate cyclase (AC) (FSK; [59]; see also [30] for non-specific actions of FSK). The data (S3B Fig) show that the AA-induced Ca^2+^ release in round spermatids was significantly inhibited by trifluoperazine (a nonspecific CaM inhibitor, also described as a ryanodine-sensitive channel agonist, [57]) but not by calmidazolium, a drug also known to be an inhibitor of CaM (both drugs appear to have CaM-independent effects on TRPV1 channels; see [60]). Hence, the results of TFP cannot be interpreted unequivocally as a result of CaM inhibition. KN62 and BIM did not produce significant changes in the AA-induced Ca^2+^ release from ICaS, strongly suggesting that CaM, CaMK or PKC are not part of the pathways activated by AA in round spermatids. Forskolin, an activator of adenylyl cyclase and hence of PKA, did not significantly affect the AA-induced release of Ca^2+^ from ICaS, suggesting that extra generation of cAMP and, likely, further PKA activation did not affect the pathways that AA triggers in these cells. However, H89, an inhibitor of PKA and other kinases [32] significantly increased the potency of AA as an inducer of Ca^2+^ release from ICaS. A similar outcome was observed for PT1, an AMPK activator.

Since the mentioned pharmacology suggested the involvement of protein phosphorylation in the AA-induced release of Ca^2+^ from ICaS in round spermatids, we used okadaic acid (OA, e.g., [39]), cantharidin (Canth, [37]) and cyclosporine A (CspA) as inhibitors of protein phosphatases and the Ca^2+^-CaM-dependent protein phosphatase calcineurin (e.g., [38]). S3C Fig shows that OA, Canth and CspA did not produce a significant effect on the ability of AA to release Ca^2+^ from ICaS.

One possible mechanism by which AA can induce the release of Ca^2+^ from ICaS could be via the activation of PLC and the generation of IP3. To test this hypothesis we used as pharmacological agents, U73122, an inhibitor of PLC (2 μM; e.g., [61]) and U73334, a described inactive analogue of U73122 (2 μM; [42]). Neither compound produced a significant effect on AA-induced Ca^2+^ release from ICaS in round spermatids (see S3D Fig). Aristolochic acid, an inhibitor of PLA2 (50 μM, e.g., [40]) significantly decreased the AA-induced changes in [Ca^2+^]_i_ in round spermatids. Thus, the effects of AA could involve a positive feedback loop, releasing AA or other PUFAs in round spermatids via the activation of PLA2.

### Pharmacological evidence of different intracellular Ca^2+^ store compartments involved in the AA-induced increase in [Ca^2+^]i in round spermatids

**Fig 5:**
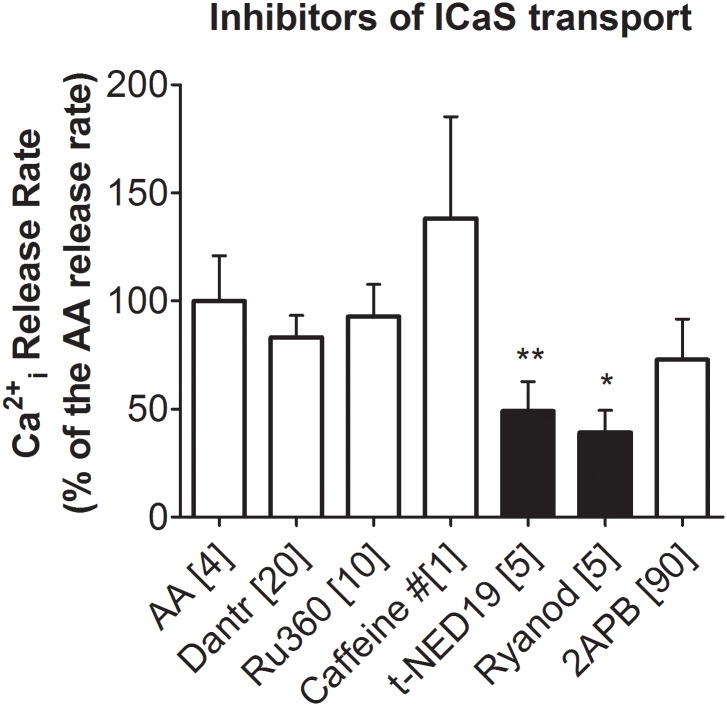
Effects of modulators of ICaS Ca^2+^ uptake or release channels. Effects of inhibitors of Ca^2+^ uptake or release channels form ICaS on the AA-induced (4 μM) release of Ca^2+^ from intracellular stores. Dantrolene (20 μM) and ryanodine (5 μM) are inhibitors of the cADPr-activated channel or Ryanodine Receptor channel (RyR). Caffeine (1 mM) is an activator of the RyR1 channel. NED19 (5 μM) is an inhibitor of the NAADP two-pore activated channel. RU360 (10 μM) is an inhibitor of mitochondrial Ca^2+^ uptake. 2APB (90 μM) is an inhibitor of IP3-activated channels. These channel modulators were added to the cells in suspension and incubated for 5 min at 33°C before AA addition, except ryanodine that was incubated with the cells for 1 h (ryanodine only binds to the open state of the RyR channel). Each bar represents the data obtained from three different cell preparations (N = 3). Significance (black bars): **, p<0.01; *, p<0.05.

As shown by Paillamanque et al. [11] and the present data, AA induced the release of Ca^2+^ form ICaS. Currently, ICaS are thought to comprise essentially three intracellular compartments: endoplasmic reticulum/nuclear envelope (including their own internal binding proteins; e.g., [62,63]), the mitochondria [64] and the acidic organelles-associated ICaS (e.g., [52]). As previously described, round spermatids have ICaS that can be depleted by treatment with thapsigargin (TSG) or cyclopiazonic acid (CPA), which are both inhibitors of the SERCA type Ca^2+^-ATPase [65–67]. Here, in order to test the type of ICaS channels that AA was activating in round spermatids we utilized a pharmacological approach using inhibitors of IP3-activated channels (2APB, [48]); of cADPribose-activated channel (RyR) (ryanodine and dantrolene, [45]); of mitochondrial Ca^2+^ uptake (Ru360, [47]) and of NAADP-activated channels (NED19, [46]). Of the mentioned inhibitors (Fig 5), ryanodine (1 h preincubation) produced approximately 60% inhibition of the AA-induced initial rate of Ca^2+^ release from ICaS. NED 19 (5 μM), the inhibitor of two-pore Ca^2+^ channels from acidic ICaS, produced an average of 50% inhibition of the AA-induced initial rate of Ca^2+^ release from ICaS.

Caffeine in a wide concentration range tested (0.1–1 mM) did not have an effect on basal [Ca^2+^]i (see also, [67]) or on the AA-induced initial rate of Ca^2+^ release from ICaS.

### NAADP-activated Ca^2+^ release from ICaS in round spermatids

**Fig 6:**
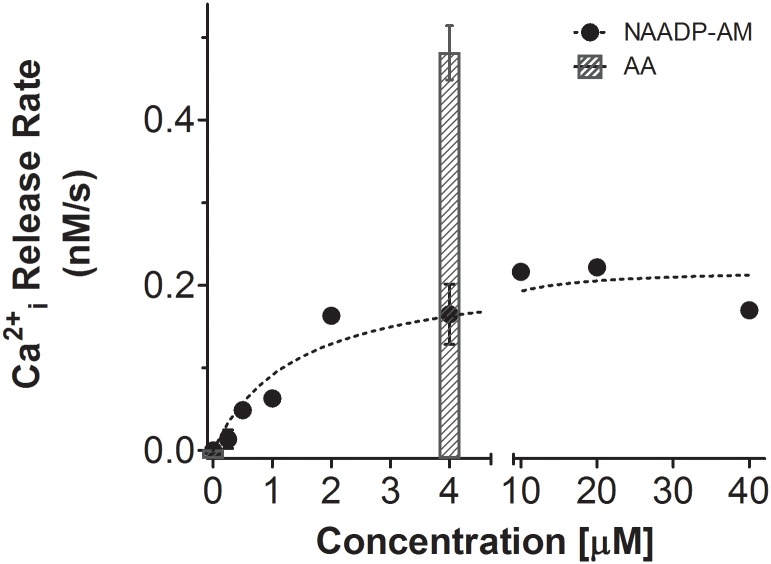
Dose-response curve of NAADP-AM on [Ca^2+^]i increase rate. Dose-response curve of NAADP-AM releasable intracellular Ca^2+^ vs NAADP-AM extracellular concentration. Cells were loaded with fura-2 and incubated in KH-lactate-EGTA media without added Ca^2+^ at 33°C. As a reference, the effect of 4 μM AA is shown as a bar. Each point represents the data obtained from three different cell preparations (N = 3).

Since NED19 induced significant inhibition of the AA-induced Ca^2+^ release from ICaS, we tested for the existence of the described NAADP-dependent stores in round spermatids. For that purpose we used a permeable analogue of NAADP [56] that has been recently utilized to demonstrate the presence and physiological role of this Ca^2+^ releasing mechanism in human sperm [68]. As shown in Fig 6, NAADP-AM was able to increase [Ca^2+^]i in round spermatids in a dose-dependent fashion with a K_0.5_ and V_max_ of 2.23±0.75 μM and 0.23±0.02 nM/s, respectively. Since the acethoxymethyl group in NAADP-AM is cleaved by intracellular esterases, the intracellular NAADP concentration is expected to be higher than the external concentration. As a reference, we show in the same graph the AA (4 μM) induced rate of [Ca^2+^]i changes. At 4 μM, NAADP-AM was able to induce an ICaS Ca^2+^ release rate approximately 35% of that induced by AA at the same concentration. Pre-incubation of the cells for 30 min with 25 μM NED19, an inhibitor of NAADP activated intracellular Ca^2+^ channels, produced on average a 95% inhibition of the NAADP-AM (1 μM)-induced Ca^2+^ release from ICaS in round spermatids.

## Discussion

Since the 1970’s, important evidence has been collected about the peculiar lipid composition of germinal cells and SCs in the testes, specifically their high content of UFAs [21,69–71]. However, the physiological significance of UFAs in the male germinal epithelium has remained unknown. In a previous work [11] we demonstrated that free UFAs can release Ca^2+^ from intracellular Ca^2+^ stores (ICaS) in pachytene spermatocytes and round spermatids. The results presented in this work strongly suggest that arachidonic acid (AA), a PUFA that can be released to the external media by SCs [4], can trigger a GPR120 (FFAR4)-like activation response in round spermatids. Thus, the dose-response curve for the AA effect on [Ca^2+^]i had a similar K_0.5_ to grifolic acid, a GPR120 agonist. However, this GPR120 agonist showed a stronger ability to induce intracellular Ca^2+^ release compared to AA. An antibody against rat C-terminal domain of GPR120 resulted in a marked labelling of interstitial cells and spermatid cells close to the lumen of the seminiferous tubules, which is consistent with a likely GPR120-mediated effect of AA on [Ca^2+^]i in round spermatids as shown here and in [11].

The effect of AA on [Ca^2+^]i was sensitive to G protein-receptor interaction, and appeared to involve ERK activation, and PLA2 activation. The possible participation of PLA2 and ERK and associated mechanisms of activation in the AA-induced release of Ca^2+^ from ICaS were not further explored in this work. Similarly, the mechanisms associated to the potentiation by activation of AMPK or inhibition of PKA on the effects of AA on ICaS release in round spermatids were not explored in this work. The effects of AA on ICaS release and the association with ERK in round spermatids are similar to those elicited by ω3 and ω6 PUFAs in Caco-2 cells, which express GPR120 but not GPR40 (FFAR1, another GPR for free medium- and large-chain fatty acids) [72]. In these cells, AA, eicosapentaenoic acid (EPA) and docosahexaenoic acid (DHA), all used at 100–200 μM (i.e., at 12–25 times higher concentration that in our work) increased [Ca^2+^]i and activated ERK1/2 in Caco-2 cells. Similarly, in HEK cells expressing GPR120 [73], oleic acid and a synthetic GPR120 agonist induced increments in [Ca^2+^]i with K_0.5_ values close to those found for grifolic acid and AA in our work. As for the possible involvement of a PLA2 in the AA-induced release of Ca^2+^ from intracellular stores suggested by our results, no literature has linked AA-Ca^2+^ and PLA2 in spermatogenic cells to our knowledge. However, Ca^2+^-dependent and Ca^2+^-independent PLA2 have been described in the testes and specifically in spermatogenic cells [74–76] and their appearance during development is correlated to the initiation of meiosis in germ cells [75].

**Fig 7:**
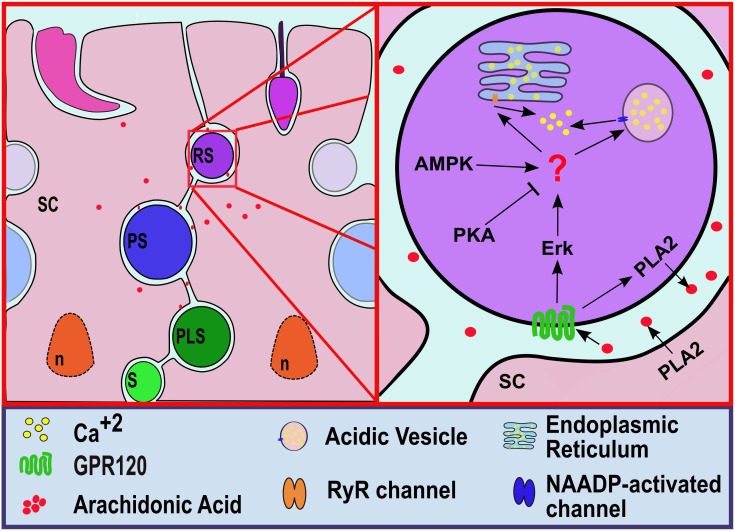
Summary diagram. Diagram showing the likely activation mechanisms of PUFA release from Sertoli cells (SC) and their mechanisms of action on spermatogenic cell intracellular Ca^2+^ stores.

As reported by Treviño *et al*. [67], spermatogenic cells express IP_3_-sensitive and cADPR-sensitive intracellular Ca^2+^ channels. In the present study, we demonstrated functionally that round spermatids also possess NAADP-sensitive intracellular Ca^2+^ channels in ICaS. Our results strongly suggest that cADPR- and NAADP-sensitive intracellular Ca^2+^ channels are involved in the AA-induced Ca^2+^ release from ICaS in round spermatids, which correlates well with the molecular infrastructure that these cells possess. Fig 7 summarizes the working model of AA-activated pathways in spermatogenic cells, the associated cell-cell interactions in the testes and their likely endocrine regulation. Thus, free fatty acid lipid signalling in the seminiferous tubules is proposed as an important mechanism for cell-cell interactions in the seminiferous epithelium associated to the regulation of spermatogenesis in the testes.

## Supporting information

S1 Fig Kinetics of grifolic acid.induced [Ca^2+^]i changes. Kinetics of grifolic acid-induced [Ca^2+^]i increase in round spermatids. At the times indicated by arrows, grifolic acid or arachidonic acid (4 μM) were added to round spermatids previously loaded with fura-2 and incubated in KH-lactate-EGTA media without added Ca^2+^ at 33°C.
(TIF)

S2 Fig Effects of pharmacological agents on basal [Ca^2+^]i. Basal effects of different pharmacological molecules used in this work on the [Ca^2+^]i changes in round spermatids. Each bar and error bar represents the average and standard deviation of data obtained for at least 3 cell preparations (N = 3).
(TIF)

S3 Fig Effects of signalling pathways modulators on AA-induced increase in [Ca^2+^]i. **A**. Effects of ERK, Akt and PI3K inhibitors on the AA-induced (4 μM) release of Ca^2+^ from intracellular stores. ERK inhibitor I (10 μM), ERK inhibitor II (5 μM), PI3K inhibitor: wortmannin (1 μM), Akt inhibitor IV (1 μM) and Akt Inhibitor V (1 μM) were added to the cells in suspension and incubated for 5 min at 33°C before AA addition. Significance: **, p<0.01. N = 3. **B**. Effects of CaM, PKC, PKA, AMPK inhibitors or activators on the AA-induced (4 μM) release of Ca^2+^ from intracellular stores. CaM inhibitors: trifluoperazine (TFP, 20 μM), calmidazolium (Cmdz, 2 μM), KN-62 (5 μM). PKC inhibitor: bisindoleylmaleimide (BIM, 2 μM). AC activator: forskolin (FSK, 5 μM). AMPK activator: PT1 (2 μM). PKA inhibitor: H89 (2 μM) were added to the cells in suspension and incubated for 5 min at 33°C before AA addition. Significance: *, p<0.05. N = 3. **C**. Effects of protein phosphatase (PPase) inhibitors on the AA-induced (4 μM) release of Ca^2+^ from intracellular stores. Okadaic acid (OA, 0.2 μM), cantharidin (Canth, 5 μM) and cyclosporine A (CspA, 5 μM) were added to the cells in suspension and incubated for 5 min at 33°C before AA addition. N = 3. **D**. Effects of phospholipases inhibitors on the AA-induced (4 μM) release of Ca^2+^ from intracellular stores. Aristolochic acid (ArA, inhibitor of PLA2, 50 μM). U73122 (inhibitor of PLC, 2 μM) and U73343 (inactive analogue of U73122, 2 μM) were added to the cells in suspension and incubated for 5 min at 33°C before AA addition. Significance: *, p<0.05. N = 3. Each bar and error bar in S3 Fig (A, B, C and D) represents the average and standard deviation of data obtained for at least 3 cell preparations (N = 3).
(TIF)
