# Supplementary figures and images for: Arachidonic acid triggers [Ca2+]i increases in rat round spermatids by a likely GPR activation, ERK signalling and ER/acidic compartments Ca2+ release
Source: PLoS One. 2017 Feb 13;12(2):e0172128. doi: 10.1371/journal.pone.0172128 (PMC5305069; doi:10.1371/journal.pone.0172128)

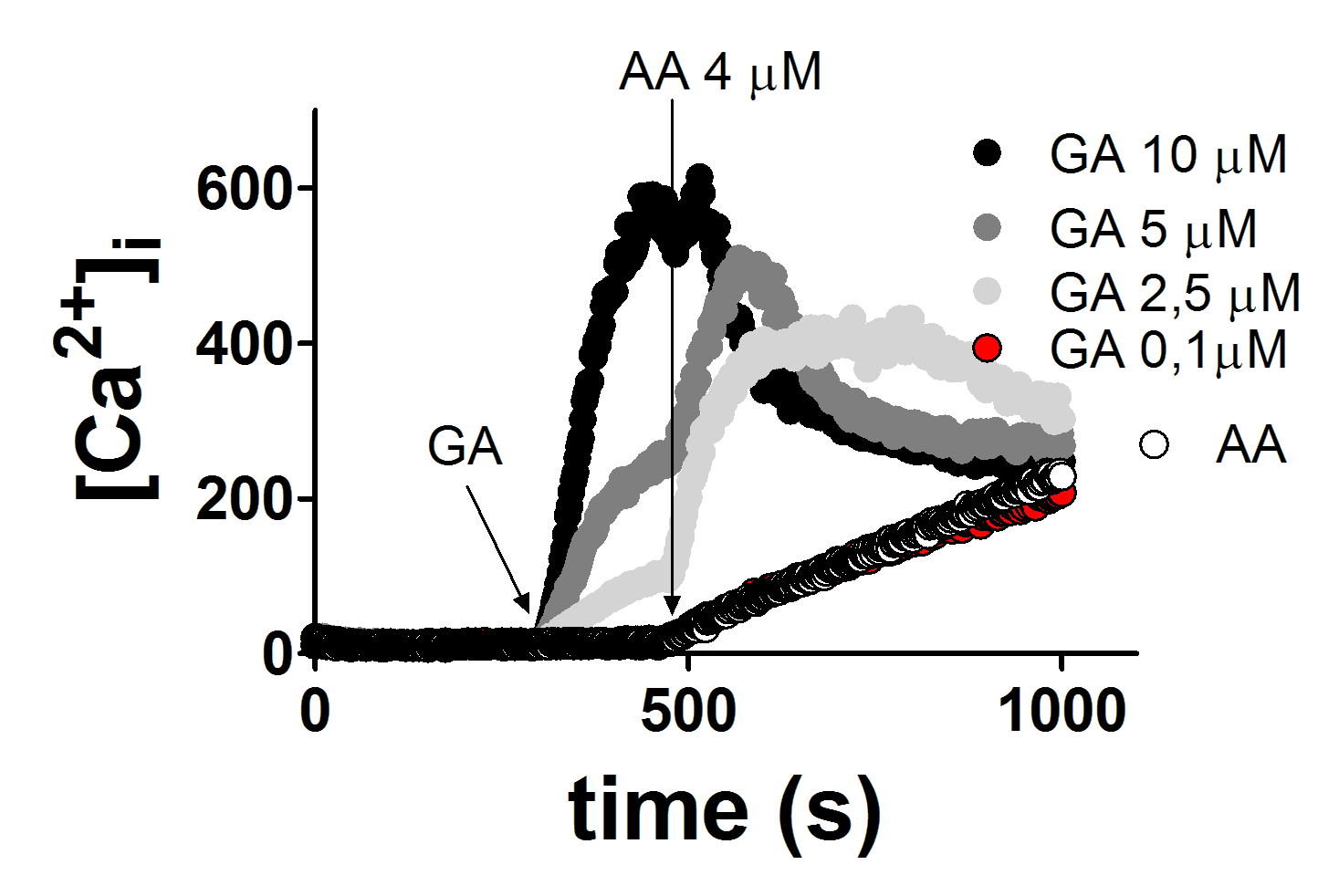

Supplement: S1 Fig — Kinetics of grifolic acid-induced [Ca2+]i increase in round spermatids. At the times indicated by arrows, grifolic acid or arachidonic acid (4 μM) were added to round spermatids previously loaded with fura-2 and incubated in KH-lactate-EGTA media without added Ca2+ at 33°C. (TIF) [file pone.0172128.s001.tif]

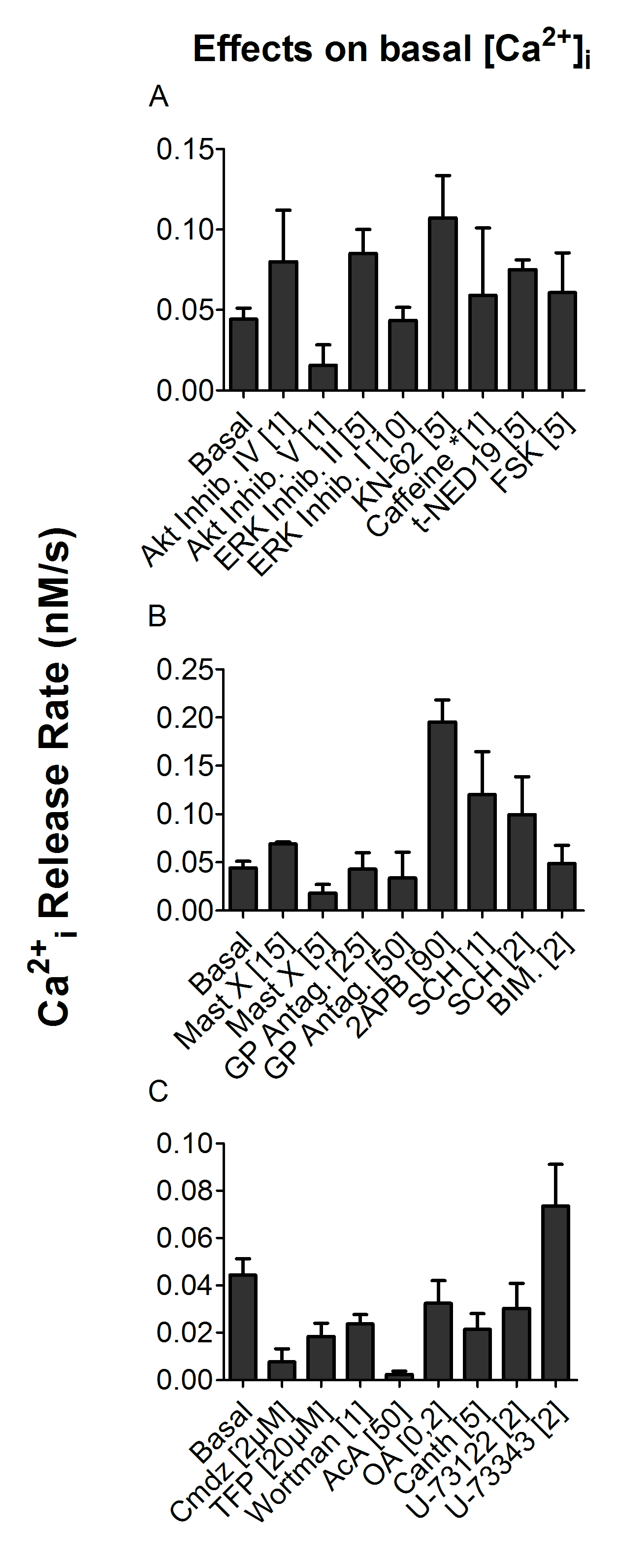

Supplement: S2 Fig — Basal effects of different pharmacological molecules used in this work on the [Ca2+]i changes in round spermatids. Each bar and error bar represents the average and standard deviation of data obtained for at least 3 cell preparations (N = 3). (TIF) [file pone.0172128.s002.tif]

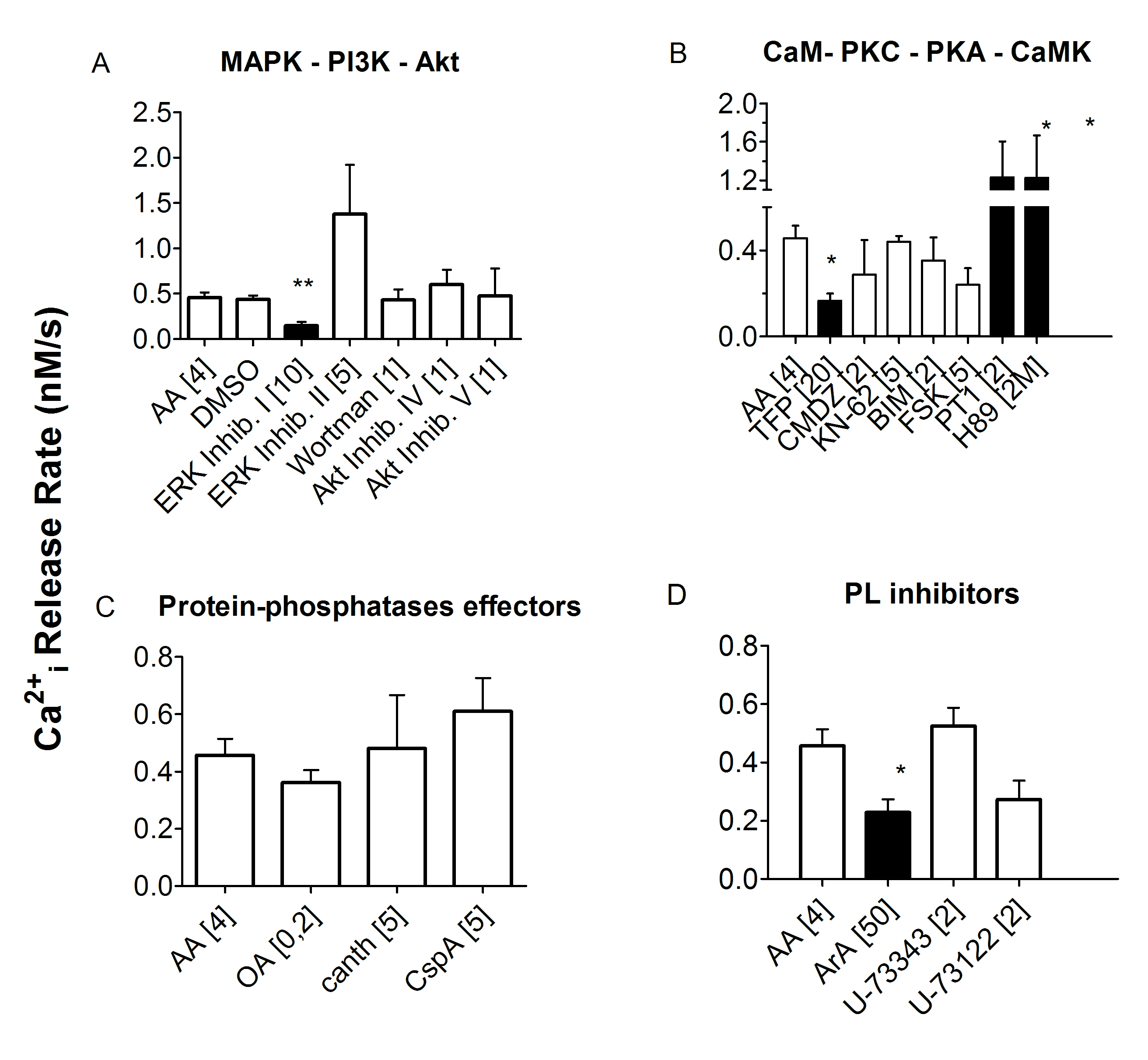

Supplement: S3 Fig — A. Effects of ERK, Akt and PI3K inhibitors on the AA-induced (4 μM) release of Ca2+ from intracellular stores. ERK inhibitor I (10 μM), ERK inhibitor II (5 μM), PI3K inhibitor: wortmannin (1 μM), Akt inhibitor IV (1 μM) and Akt Inhibitor V (1 μM) were added to the cells in suspension and incubated for 5 min at 33°C before AA addition. Significance: **, p<0.01. N = 3. B. Effects of CaM, PKC, PKA, AMPK inhibitors or activators on the AA-induced (4 μM) release of Ca2+ from intracellular stores. CaM inhibitors: trifluoperazine (TFP, 20 μM), calmidazolium (Cmdz, 2 μM), KN-62 (5 μM). PKC inhibitor: bisindoleylmaleimide (BIM, 2 μM). AC activator: forskolin (FSK, 5 μM). AMPK activator: PT1 (2 μM). PKA inhibitor: H89 (2 μM) were added to the cells in suspension and incubated for 5 min at 33°C before AA addition. Significance: *, p<0.05. N = 3. C. Effects of protein phosphatase (PPase) inhibitors on the AA-induced (4 μM) release of Ca2+ from intracellular stores. Okadaic acid (OA, 0.2 μM), cantharidin (Canth, 5 μM) and cyclosporine A (CspA, 5 μM) were added to the cells in suspension and incubated for 5 min at 33°C before AA addition. N = 3. D. Effects of phospholipases inhibitors on the AA-induced (4 μM) release of Ca2+ from intracellular stores. Aristolochic acid (ArA, inhibitor of PLA2, 50 μM). U73122 (inhibitor of PLC, 2 μM) and U73343 (inactive analogue of U73122, 2 μM) were added to the cells in suspension and incubated for 5 min at 33°C before AA addition. Significance: *, p<0.05. N = 3. Each bar and error bar in S3 Fig (A, B, C and D) represents the average and standard deviation of data obtained for at least 3 cell preparations (N = 3). (TIF) [file pone.0172128.s003.tif]
